# Activation studies of the β-carbonic anhydrases from *Malassezia restricta* with amines and amino acids

**DOI:** 10.1080/14756366.2020.1743284

**Published:** 2020-03-27

**Authors:** Andrea Angeli, Sonia Del Prete, Cynthia Ghobril, Julien Hitce, Cécile Clavaud, Xavier Marrat, William A. Donald, Clemente Capasso, Claudiu T. Supuran

**Affiliations:** a Dipartimento Neurofarba, Sezione di Scienze Farmaceutiche e Nutraceutiche, Università degli Studi di Firenze, Sesto Fiorentino (Florence), Italy; b Department of Biology, Agriculture and Food Sciences, Institute of Biosciences and Bioresources, CNR, Napoli, Italy; c L’Oréal Research and Innovation, Aulnay-sous-Bois, France; d School of Chemistry, University of New South Wales, Sydney, New South Wales, Australia

**Keywords:** Carbonic anhydrase, activator, β-class enzyme, pathogenic fungi, amine, amino acid

## Abstract

The β-carbonic anhydrase (CA, EC 4.2.1.1) from the genome of the opportunistic pathogen *Malassezia restricta* (MreCA), which was recently cloned and characterised, herein has been investigated for enzymatic activation by a panel of amines and amino acids. Of the 24 compounds tested in this study, the most effective MreCA activators were L-adrenaline (K_A_ of 15 nM), 2-aminoethyl-piperazine/morpholine (K_A_s of 0.25–0.33 µM), histamine, L-4-amino-phenylalanine, D-Phe, L-/D-DOPA, and L-/D-Trp (K_A_s of 0.32 − 0.90 µM). The least effective activators were L-/D-Tyr, L-Asp, L-/D-Glu, and L-His, with activation constants ranging between 4.04 and 12.8 µM. As MreCA is involved in dandruff and seborrhoeic dermatitis, these results are of interest to identify modulators of the activity of enzymes involved in the metabolic processes of such fungi.

## 1. Introduction

Carbonic anhydrases (CAs; EC 4.2.1.1) are present in most organisms investigated to date^1–5^, with eight genetically distinct classes of such enzymes, the α-, β-, γ-, δ-, ζ-, η-, θ-, and ι-CA classes being encoded in the genome of various organisms^6–8^. They all catalyse the simple but fundamental interconversion reaction between carbon dioxide and bicarbonate, with the comcomitant generation of hydronium ions:

$${\text{CO}}_{2}+{2H}_{2}O\;⇌\;{\text{HCO}}_{3}^{-}+H_{3}O^{+}$$

α-CAs are Zn^2+^ metalloproteins expressed in vertebrates, fungi, protozoa, algae, plants and prokaryotes^4–9^. The β-CAs are also Zn^2+^ enzymes and they are present in bacteria, fungi, protozoa and chloroplasts of mono-/dicotyledon plants^4^^,^^5^. γ-CAs are probably Zn^2+^ or Fe^2+^ enzymes, although it has been shown they are also active with Co^2+^ within their active site, and are present in archaea, bacteria and plants^3^^,^^4^. Limited information is known about δ-CAs, which are zinc- or cobalt-containing enzymes present in marine diatoms^7^^,^^10^. The ζ-CAs are active with Cd^2+^ or Zn^2+^ at their active site, and were also identified in marine diatoms^11^. η-CA are Zn^2+^ metalloproteins identified in *Plasmodium* spp. and other protozoans^12^. The recently identified θ- and ι-CAs are also present in marine diatoms^11^^,^^13^, and the latter class is also expressed in bacteria and are likely Mn(II) metalloenzymes, as recently reported^13^.

Various classes of inhibitors of these enzymes, mainly targeting mammalian CAs, are in clinical use as diuretics, antiglaucoma, antiepileptic or antiobesity agents for decades, whereas their use as anticancer agents started to be contemplated only in the last decade^1^^,^^2^^,^^6^^,^^14–18^. There has also been recent interest in inhibiting CAs in various pathogenic bacteria to develop anti-infective applications^6–8^. These diverse applications are due to the fact that at least 15 different α-CA isoforms are present in humans, being involved in critical physiological and pathological processes^14–18^.

Activation studies of various classes of CAs, among which the β-, γ-, δ-, ζ-, and η-CA classes were explored only recently, and only with two classes of modulators of activity, the amines and the amino acids^3^^,^^19^. The catalytic mechanism of these enzymes is also well understood and explains also their activation mechanism^3^. A metal hydroxide species present in the active site of these enzymes acts as a strong nucleophile (at physiologic pH) for converting the CO_2_ to bicarbonate, which is thereafter coordinated to the catalytic metal ion. This adduct is not very stable and its reaction with an incoming water molecule leads to the liberation of bicarbonate in solution and generation of an acidic form of the enzyme incorporating an M^2+^(OH_2_) species at the metal centre, which is catalytically ineffective for the hydration of CO_2_^1–3^_._ To generate the nucleophilic M^2+^(OH^–^) species, a proton transfer reaction occurs, which determines the rate for the catalytic cycle in many of these types of very efficient enzymes. For many α-CAs this step is assisted by a proton shuttle residue, which is His64 in most mammalian isoforms^20^. This is one of the few residues in α-CAs possessing a flexible conformation, with an inward (*in* conformation) and outward (*out)* conformation. For this reason, the imidazole moiety of this histidine, with a p*K*_a_ of 6.0–7.5 (depending on the isoform^3^) is an appropriate proton shuttling residue which transfers the proton from the metal coordinated water to the reaction medium, in a crucially important and rate-determining step of the catalytic cycle^1–3^. The process can also be assisted by endogenous molecules, which bind within the enzyme active site, as proven by X-ray crystallography and other techniques, which have been termed CA activators (CAAs)^19^. Such activators facilitate the proton transfer reactions between the metal ion centre and the external medium by an alternative pathway to the proton shuttle residue. The two reactions of the CA catalytic cycle are shown by Equations (1) and (2), with the deprotonation of zinc-bound water being the rate-determining step (Equation (2)^19^^,^^21^. This leads to the generation of the active form of the enzyme^3^^,^^19^^,^^22^:

(1) $$EZn^{2+}-OH^{-}+CO_{2}⇌EZn^{2+}-HCO_{3}^{-}\overset{+H_{2}O}{⇌}EZn^{2+}-OH_{2}+HCO_{3}^{-}$$

(2) $$EZn^{2+}-OH_{2}⇌EZn^{2+}-OH^{-}+H^{+}-\mathrm{rate}\;\mathrm{determining}\;\mathrm{step}-$$

In the presence of an activator molecule “A”, Equation (2) becomes Equation (3); that is, in the enzyme-activator complex the proton transfer reaction is no longer intermolecular but intramolecular, and thus favoured^3^^,^^19^:

(3) $${\text{EZn}}^{2+}-{\text{OH}}_{2}+A⇌[{\text{EZn}}^{2+}-{\text{OH}}_{2}-\;A]\;⇌\;[{\text{EZn}}^{2+}-{\text{HO}}^{-}-{\;\text{AH}}^{+}]\;⇌{\text{EZn}}^{2+}-{\text{HO}}^{-}+{\;\text{AH}}^{+}$$

### Enzyme–activator complexes

CAAs were recently demonstrated to have potential pharmacologic applications^23^, as the activation of mammalian enzymes was shown to enhance cognition and memory in experimental animals^23^^a,b^, whereas its inhibition had the opposite effect^14^.

**Figure 1.**
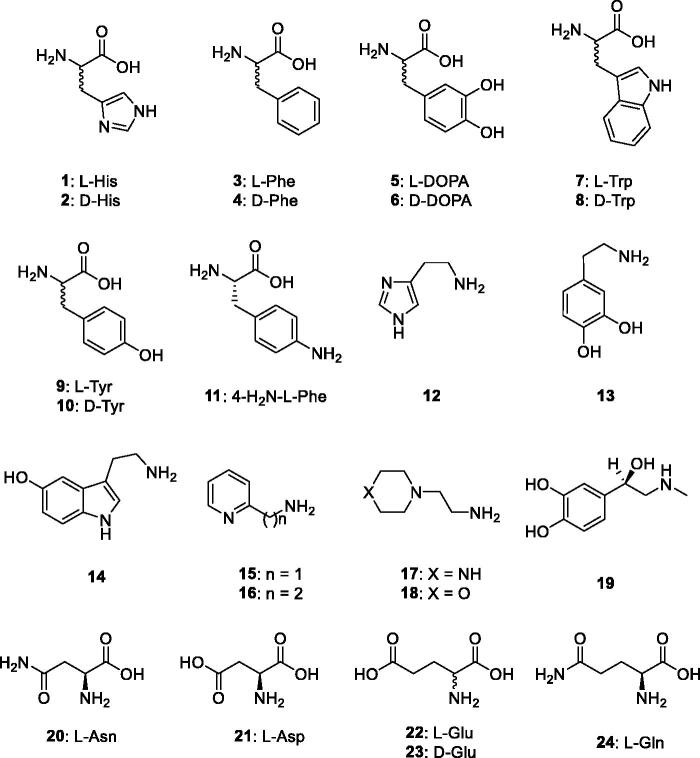
Amino acids and amines **1–24** investigated as CAAs in the present article.

The activation of CAs from pathogenic bacteria may also be relevant for understanding the factors governing virulence and colonisation of the host, because pH in the tissues surrounding the pathogens likely plays a key role in such processes^3^^,^^5^ and many compounds that are CAAs (biogenic amines and amino acid derivatives) are abundant in such tissues. Considering such evidence, in this study we report an activation study with amines and amino acids (compounds **1–24**, Figure 1) of the β-CA recently reported and characterised biochemically from the dandruff producing organism *Malassezia restricta*^24^.

## 2. Materials and methods

### 2.1. Enzymes production and purification

The protocol described in the previous works^24^ has been used to obtain purified recombinant MreCA.

### 2.2. Ca activity/activation measurements

An Sx.18Mv-R Applied Photophysics (Oxford, UK) stopped-flow instrument has been used to assay the catalytic activity of various CA isozymes for CO_2_ hydration reaction^25^. Phenol red (at a concentration of 0.2 mM) was used as indicator, working at the absorbance maximum of 557 nm, with 10 mM Hepes (pH 7.5, for α-CAs)^26–29^ or TRIS (pH 8.3, for β-CAs)^30–33^ as buffers, 0.1 M NaClO_4_ (for maintaining constant ionic strength), following the CA-catalyzed CO_2_ hydration reaction for a period of 10 s at 25 °C. The CO_2_ concentrations ranged from 1.7 to 17 mM for the determination of the kinetic parameters and inhibition constants. For each activator at least six traces of the initial 5–10% of the reaction have been used for determining the initial velocity. The uncatalyzed rates were determined in the same manner and subtracted from the total observed rates. Stock solutions of activators (at 0.1 mM) were prepared in distilled-deionized water and dilutions up to 1 nM were made thereafter with the assay buffer. Enzyme and activator solutions were pre-incubated together for 15 min prior to assay, in order to allow for the formation of the enzyme–activator complexes. The activation constant (K_A_), defined similarly with the inhibition constant K_I_, can be obtained by considering the classical Michaelis–Menten equation (Equation (4), which has been fitted by non-linear least squares by using PRISM 3:

(4) $$v=v_{\text{max}}/\left\{1+\left(K_{M}/\left[S\right]\right)\left(1+{\left[A\right]}_{f}/K_{A}\right)\right\}$$

where [A]_f_ is the free concentration of activator.

Working at substrate concentrations considerably lower than K_M_ ([S] ≪K_M_), and considering that [A]_f_ can be represented in the form of the total concentration of the enzyme ([E]_t_) and activator ([A]_t_), the obtained competitive steady-state equation for determining the activation constant is given by Equation (5):

(5) $$v=v_{0}\cdot K_{A}/\left\{K_{A}+{\left({\left[A\right]}_{t}-0.5\{\left({\left[A\right]}_{t}+{\left[E\right]}_{t}+K_{A}\right)-{\left({\left[A\right]}_{t}+{\left[E\right]}_{t}+K_{A}\right)}^{2}-4{\left[A\right]}_{t}.{\left[E\right]}_{t}\right)}^{1/2}\}\right\}$$

where v_0_ represents the initial velocity of the enzyme-catalyzed reaction in the absence of activator^3^^,^^30–35^. This type of approach to measuring enzyme-ligand interactions is in excellent agreement with recent results from native mass spectrometry measurements^36^.

### 2.3. Reagents

Amines and amino acid derivatives **1–24** were obtained in the highest purity that was available commercially from Sigma-Aldrich (Milan, Italy).

## 3. Results and discussion

We measured the kinetics constants (k_cat_ and K_M_) of the recently described β-CA from *M. restricta*, MreCA, for comparison to those of the thoroughly studied human (h) CA isoforms hCA I and II, belonging to the α-CA class (Table 1). The experiments were also performed in the presence of 10 μM L-Trp as activator.

**Table 1.** Activation of hCA I, II and MreCA with L-Trp, at 25 °C, for the CO_2_ hydration reaction^25^.

Enzyme	k_cat_^a^	K_M_^a^	(k_cat_)_L-Trp_^b^	K_A_^c^(μM)
	(s ^− 1^)	(mM)	(s^−1^)	L-Trp
hCA I^d^	2.0 × 105	4.0	3.4 × 105	44.0
hCA II^d^	1.4 × 106	9.3	4.9 × 106	27.0
MreCA^e^	1.06 × 106	9.9	9.6 × 106	0.32

^a^Observed catalytic rate without activator. K_M_ values in the presence and the absence of activators were the same for the various CAs (data not shown).

^b^Observed catalytic rate in the presence of 10 μM activator.

^c^The activation constant (K_A_) for each enzyme was obtained by fitting the observed catalytic enhancements as a function of the activator concentration^25^. Mean from at least three determinations by a stopped-flow, CO_2_ hydrase method^25^. Standard errors were in the range of 5–10% of the reported values (data not shown).

^d^Human recombinant isozymes, from work by Capasso and Supuran^32^.

^e^Fungal recombinant enzyme, this work.

The data in Table 1 indicates that the presence of L-Trp does not change the K_M_ both for the two enzymes belonging to the α-class (hCA I/II) as well as for MreCA, a situation also observed for all CA classes for which CA activators have been investigated so far^3^^,^^29–33^. In fact, as proven by kinetic and crystallographic data^3^^,^^20^, the activator binds in a diverse binding region within the active site of the substrate binding site. Thus, the activator does not influence K_M_ but has an effect only on the k_cat_. Indeed, a 10 µM concentration of L-Trp leads to a 9-fold enhancement of the kinetic constant of MreCA compared to the same parameter in the absence of the activator (Table 1). For hCA I and II, the enhancement of the kinetic constant in the presence of L-Trp was rather modest, as these enzymes show a weaker affinity for this activator (Table 1). On the other hand, L-Trp has a submicromolar affinity for MreCa which explains its potent activating effect (see discussion later in the text).

Thus, an entire range of amines and amino acids, of types **1–24**, were tested for their efficacy as MreCA activators (Table 2). These compounds were also investigated earlier^3^ for their activating properties against hCAs and many enzymes from pathogenic organisms, as reported previously^26–33^. The following structure–activity relationship (SAR) for the activation of MreCA with compounds **1–24** has been documented considering the data in Table 2:

**Table 2.** Activation constants of hCA I, hCA II and MreCA with amino acids and amines **1–24** by a stopped-flow CO_2_ hydrase assay^25^.

No.	Compound	K_A_ (μM)^a^		
		hCA I^b^	hCA II^c^	MreCA^c^
**1**	L-His	0.03	10.9	12.8
**2**	D-His	0.09	43	1.84
**3**	L-Phe	0.07	0.013	2.69
**4**	D-Phe	86	0.035	0.76
**5**	L-DOPA	3.1	11.4	0.87
**6**	D-DOPA	4.9	7.8	0.70
**7**	L-Trp	44	27	0.32
**8**	D-Trp	41	12	0.89
**9**	L-Tyr	0.02	0.011	4.15
**10**	D-Tyr	0.04	0.013	7.83
**11**	4-H_2_N-L-Phe	0.24	0.15	0.61
**12**	Histamine	2.1	125	0.90
**13**	Dopamine	13.5	9.2	2.71
**14**	Serotonin	45	50	0.82
**15**	2-Pyridyl-methylamine	26	34	0.34
**16**	2–(2-Aminoethyl)pyridine	13	15	2.13
**17**	1–(2-Aminoethyl)-piperazine	7.4	2.3	0.25
**18**	4–(2-Aminoethyl)-morpholine	0.14	0.19	0.33
**19**	L-Adrenaline	0.09	96.0	0.015
**20**	L-Asn	11.3	>100	0.93
**21**	L-Asp	5.20	>100	4.04
**22**	L-Glu	6.43	>100	5.26
**23**	D-Glu	10.7	>100	4.70
**24**	L-Gln	>100	>50	0.90

^a^Mean from three determinations by a stopped-flow, CO_2_ hydrase method^25^. Standard errors were in the range of 5–10% of the reported values (data not shown).

^b^Human recombinant isozymes, from the work by Supran and collegues^3^.

^c^Fungal recombinant enzyme, this work.

- The compounds which showed the least effective for activating MreCA were L-His, L-/D-Tyr, L-Asp, and L-/D-Glu, with activation constants ranging between 4.04 and 12.8 µM. These compounds belong to a rather heterogeneous group of amino acids, with both deprotonated (Asp, Glu), neutral (Tyr) and protonated (His) side chains at pH 7.4. On the other hand, it seems that in some cases the enantiomer is relevant for this activity, if one compares the differences in K_A_ between L and D-His, with the last compound being 6.95 times a better activator compared to its diastereoisomer (Table 2).
- Compounds possessing a medium activating effect were D-His, L-Phe, dopamine and 2-(2aminoethyl)pyridine (derivative **16**), which showed activation constants in the range of 1.84–2.71 μM (Table 1). Again, small structural changes, as in the pair of compounds **15**/**16**, leads to drastic changes of activity. The two compounds only differ by a CH_2_ group, but **15** is 6.26 times a more effective activator compared to **16**.
- The effective, submicromolar CAAs against MreCA detected here were D-Phe, L-/D-DOPA, and L-/D-Trp, 4-amino-L-phenylalanine, 2-aminoethyl-piperazine/morpholine, histamine, serotonin, some pyridyl-alkylamines, L-Gln, and L-Asn, with K_A_s of 0.25–0.93 µM. L-adrenaline, with a K_A_ of 15 nM, was the most effective among all compounds investigated here for the activation of MreCA (Table 2).
- The activation profile of this fungal enzyme with amino acids and amines is very different from that of the human isoforms hCA I and II, with only L-Asn and L-Gln showing some selectivity for the activation of the fungal versus the human enzymes.

## 4. Conclusions

CAs were shown to be involved in metabolic and signalling pathways in fungi, including pathogenic ones, and this mechanism has been proposed to be exploited for the development of antifungals with different mechanisms of action compared to the clinically used agents, for which extensive drug resistance has been documented^7^^,^^24^^,^^34^. Indeed, in an animal model of dandruff provoked by *M. globosa*, a related species to *M. restricta*, it has been shown that β-CA inhibition with sulphonamides has a potent antifungal effect^35^. However, there are no studies to date on the role of CAAs on the life cycle of fungal pathogens. Considering the fact that amines and amino acids as those investigated here are found in high concentrations in many tissues, our present finding may be relevant for a better understanding of processes connected with infectivity and growth of fungal pathogens. L-adrenaline was observed to be the best MreCA activator. Is it a coincidence that stress, i.e. higher circulating amounts of catecholamines such as L-adrenaline, is associated with a worsening of seborrhoeic dermatitis and dandruff?
